# Live fast, die young? Day‐ and night‐warming affect the growth, survivorship, and behavior of caterpillars in the field

**DOI:** 10.1002/ecy.70150

**Published:** 2025-07-03

**Authors:** Louie H. Yang, Elizabeth G. Postema, Heran Arefaine, Fernanda Y. Cohoon, Emma A. Deen, Yvonne L. Durand, Gwendolyn I. Erdosh, Hailey Ma, Courtney N. Mausling, Sarah Solís, Madeline R. Wilson

**Affiliations:** ^1^ Department of Entomology and Nematology University of California Davis California USA; ^2^ Graduate Group in Animal Behavior University of California Davis California USA; ^3^ Department of Evolution and Ecology University of California Davis California USA; ^4^ Present address: Field Museum of Natural History Chicago Illinois USA

**Keywords:** climate change, diel activity, ectotherms, handwarmers, heatwaves, metabolic meltdown, night‐warming, nocturnal behavior, *Pieris rapae*, temporal thermal refuges, thermal performance, thermal stress

## Abstract

While both daytime and nighttime temperatures are increasing with climate change, few studies have experimentally investigated their differential effects under field conditions. We conducted a factorial field experiment examining how day‐ and night‐warming impact the growth, survivorship, and behavior of cabbage white caterpillars (*Pieris rapae*). In this experiment, the night‐warming only treatment showed the highest rates of caterpillar growth, but also showed the highest mortality, the shortest maximum caterpillar lengths, the least accumulated herbivory, and reduced pupation. Caterpillars in the treatments that were not warmed during the day showed daytime‐shifted growth, and caterpillars in the combined day‐ and night‐warming treatment showed strongly night‐shifted herbivory. Both biotic (e.g., predation risk) and abiotic (e.g., thermal) factors could have contributed to these results. Broadly, these results show the importance of temperature‐mediated behavioral changes in diel activity for caterpillar development and survival. These results also support the emerging hypotheses that periods of reduced activity may be important for successful development, that warmer nighttime conditions could limit a temporal thermal refuge for caterpillars, and that increasing temperatures could increase the likelihood of metabolic meltdown. This experiment also illustrates the value of field studies to provide insights into how ectotherms might respond to ongoing climate change.

## INTRODUCTION

Increased nighttime warming is an important consequence of climate change (Davy et al., 2017; IPCC, 2021) and nighttime temperatures have increased faster than daytime temperatures over the past half‐century (Davy et al., 2017). This pattern of asymmetrical day/night‐warming is likely to be stronger in some regions than others (Cox et al., 2020), and may even have reversed somewhat recently (Zhong et al., 2023). However, it is clear that the frequency, duration, and magnitude of warmer nighttime temperatures have increased substantially around the world, and that this trend is expected to continue (Seneviratne et al., 2021). However, the study of ecology at night remains a persistent challenge (Gaston, 2019). The importance of this “nocturnal problem” has been recognized for nearly a century (Elton, 1927; Gaston, 2019), despite increasing awareness of the importance of nighttime species interactions. For example, a recent meta‐analysis indicated that worldwide insect activity is 31% higher at night than during the day (Wong & Didham, 2024).

Despite rising nighttime temperatures and likely effects on species interactions, relatively few studies have isolated the effects of nighttime warming (Speights et al., 2017). Both lab and field studies have long manipulated constant temperatures (e.g., Braby & Lyonns, 2003; Zalucki, 1982; Zhang et al., 2015), daytime temperatures (e.g., Birkemoe et al., 2016; Kingsolver et al., 2021; Ma, Hoffmann, & Ma, 2015) or overall warming (e.g., Kingsolver et al., 2015; Ma et al., 2017). It has commonly been shown that insects reared under fluctuating (as opposed to constant) temperature regimes can continue to develop under higher maximum daytime temperatures (Kingsolver et al., 2015; Nail et al., 2015; Zhao et al., 2014, but see also Paaijmans et al., 2013; Stocker et al., 2024). These findings support the general hypothesis that cooler nighttime temperatures allow ectotherms to repair thermal damage accumulated during the day (Bai et al., 2019; Colinet et al., 2015; González‐Tokman et al., 2020; Ma et al., 2017; Ørsted et al., 2022; Speights et al., 2017), emphasizing the importance of measuring performance in the context of realistically variable temperature regimes (Bai et al., 2019; Colinet et al., 2015; Kingsolver et al., 2015; Ma et al., 2021). Recent studies have also highlighted the importance of separating the developmental and survival components of thermal performance (Abarca et al., 2024; Paaijmans et al., 2013; Whitney‐Johnson et al., 2005; Zhao et al., 2014), and considering thermal performance over multiple life stages (Chiu et al., 2015; Sinclair et al., 2016).

Our emerging understanding of thermal stress and repair suggests the importance of experiments that separately consider the implications of day‐ and night‐warming under more realistic conditions (Bai et al., 2019; Colinet et al., 2015; González‐Tokman et al., 2020; Ma et al., 2017; Speights et al., 2017). For example, the microclimates available in complex habitats could provide thermal refuges (Barton & Schmitz, 2018; Nice & Fordyce, 2006; Pincebourde & Woods, 2020; Vives‐Ingla et al., 2023), and studies that consider other members of the surrounding community could allow for more complex interactions (Barton & Schmitz, 2018; Kharouba & Yang, 2021; Lemoine et al., 2017; Moreira et al., 2024). In particular, field experiments could allow organisms to express changes in diel activity, habitat selection, or other behaviors that may not be apparent in the lab (Barton & Schmitz, 2018; Nielsen & Papaj, 2015; Vives‐Ingla et al., 2023). However, few experimental studies have examined the ecological effects of daytime and nighttime warming in the field (Speights et al., 2017, but see Barton & Schmitz, 2018). In some cases, methodological limitations could explain the lack of nighttime warming manipulations, for example, field studies that use passive solar warming (Speights et al., 2018). In other cases, the emphasis on daytime temperatures reflects an intentional focus on the frequency, duration and magnitude of extreme high daytime temperatures (Chiu et al., 2015; Kingsolver et al., 2021; Ma, Hoffmann, & Ma, 2015; Ma, Rudolf, & Ma, 2015; York & Oberhauser, 2002). However, these previous studies support the need to examine nighttime warming effects in field experiments.

We conducted an experiment to measure the effects of day‐ and night‐warming on a free‐living insect ectotherm throughout its development in the field. We asked: how do day‐ and night‐warming affect the growth, development, survival, behavior, and interactions of our focal species? Our expectation was that increasing daytime temperatures would increase exposure to stressful thermal conditions, while warmer nighttime temperatures would accelerate development within a thermally permissive range, consistent with a previous lab study (Bai et al., 2019). However, an alternative hypothesis was that increased nighttime temperatures would reduce opportunities for thermal repair, resulting in negative effects on survivorship, consistent with another lab study (Zhao et al., 2014). While there are long‐standing hypotheses about the effects of temperature on size at maturity (the temperature‐size rule, Angilletta et al., 2004; Atkinson, 1994) and the effects of growth rate on natural enemy risk (the slow growth‐high mortality hypothesis, Benrey & Denno, 1997), empirical patterns have been more variable and complex than the simple predictions of either hypothesis (Angilletta & Dunham, 2003; Chen & Chen, 2018; Fordyce & Shapiro, 2003; Walters & Hassall, 2006), and neither hypothesis explicitly considers day and night temperatures. One study that did examine day‐ and night‐warming in the field indicated the importance of thermal refuges at the plant and insect scale (Barton & Schmitz, 2018). Consistent with these findings, we hypothesized that caterpillars experiencing thermal stress during the day might shift their diel activity periods, potentially seeking temporal thermal refuges by becoming more active (increasing herbivory or growth) at night. However, we also anticipated that varied responses of the surrounding community (plants, competitors and natural enemies) could create more complex effects on the behavior, growth, and survivorship of our focal caterpillar species. Thus, this study aims to bridge the gap between controlled laboratory studies and community‐level field studies by examining the effects of day‐ and night‐warming on the growth, survivorship, and behavior of individual caterpillars in the field.

## METHODS

We conducted a fully crossed factorial field experiment to measure the independent and interactive effects of day‐ (present vs. absent) and night‐warming (present vs. absent) on the growth, survivorship and behavior of cabbage white caterpillars (*Pieris rapae*), specialist herbivores of mustard plants (Brassicaceae). Like most insects, cabbage whites are “thermo‐conforming” ectotherms whose internal temperature reflects their microclimatic conditions (Kingsolver, 2000). The cabbage white (*P. rapae*) is native to Europe, and is now widely distributed throughout North America, Asia and Australia where they commonly experience wide seasonal and diel temperature variation. Caterpillars feed on a wide range of wild and domesticated Brassicaceae, and generally do not leave their host plants until just prior to pupation (Kingsolver, 2000). Cabbage whites commonly have multiple generations per year during the warm season, and diapause as pupae during the cool season.

### Field site establishment

We created a 12 × 10 grid of plots spaced two meters apart in a level grassland field site (38.52978° N, 121.78163° W) on the University of California, Davis campus near both agricultural research fields and the Putah Creek Riparian Reserve (Appendix S1: Figure S1). The plant community at this site included a combination of non‐native annual grasses (e.g., *Avena barbata* and *Bromus hordeaceus*) and forbs (*Vicia sativa*), as well as native California species (e.g., *Achyrachaena mollis* and *Eschscholzia californica*). Each plot was centered around a focal host plant, established in 3.8 cm diameter × 14 cm deep plant containers (Ray Leach SC7U Cone‐tainers), which were placed into a matching dibble hole so that the soil in the container was approximately flush with the ground. Around each focal host plant, we set up a cylindrical, open‐topped, insulated thermal shroud (~20 cm diameter × 35 cm tall) constructed of a silver reflective Mylar bubble sheet attached to wooden dowels staked into the ground around each hole. This thermal shroud was designed to retain heat from our experimental manipulation, while remaining open on the top and bottom to allow the free movement of air and organisms.

### Host plant establishment

We established radish host plants (Brassicaceae: *Raphanus sativus*) at the Orchard Park Greenhouse Facility at the University of California, Davis. These plants were germinated on a greenhouse mist bench on April 26, 2022, transferred to an unmisted bench maintained at 24°C on May 5, 2022 and then to an open 50% shade lathe house to harden on May 13, 2022. Prior to deployment in the field, plants were watered by overhead spray. After deployment, each plant container was carefully removed from the plot and bottom‐watered by soaking to saturation twice a day.

### Herbivore collection and neonate production

We collected eight female cabbage white butterflies (*P. rapae*) using aerial nets in Davis, CA, USA on May 13, 2022. To induce oviposition, these females were provided with simulated nectar (cotton rolls soaked with fruit punch Gatorade) and oviposition substrates (*Brassica oleracea* cabbage leaves) in mesh‐topped 1 L plastic containers (BugDorm, MegaView Science Co.) held in an environmental chamber (MLR‐351H, Sanyo Corp.) under day:night temperatures of 30:20°C at 60% relative humidity and a 11:13 h light:dark photoperiod. We collected eggs from five females and allowed them to develop undisturbed on cabbage leaf fragments in 14.5 cm diameter vented plastic petri dishes before they were transferred as neonates to their host plants. We transferred 80 neonate caterpillars (16 from each of five maternal lines) onto individual 10–15‐cm‐tall radish plants with paintbrushes on May 19, 2022. These caterpillars were allowed to establish on their host plants in the lab for approximately 24 h before being introduced into the field.

### Warming treatments

We randomly assigned 80 plots to a 2 × 2 factorial combination of day‐ and night‐warming: no day‐warming and no night‐warming (“C/C,” *N* = 20), no day‐warming with night‐warming present (“C/W,” *N* = 20), day‐warming present with no night‐warming (“W/C,” *N* = 20), and combined day‐warming and night‐warming (“W/W,” *N* = 20). For the day‐warming plots, two 28.5 g (5 × 9 cm) single‐use, air‐activated heat packs (Hot Hands, Kobayashi Healthcare International, Dalton, GA, USA) were attached to wooden stakes approximately 10 cm from the focal plant, and 15 cm from the ground between 6:00 and 9:00 AM each day. For the night‐warming plots, heat packs were deployed between 6:00 and 9:00 PM each day. Each heat pack produced a consistent amount of heat for approximately 10 h via exothermic oxidation when exposed to air. Previously expended, inactive heat packs were deployed in unwarmed plots to control for the presence of a heat pack.

Thirty‐five Thermochron iButton temperature loggers (DS1922L, Maxim Integrated Products, San Jose, CA, USA) were randomly assigned to plots (C/C, *N* = 8; C/W, *N* = 8; W/C, *N* = 9; W/W, *N* = 9) in order to record ambient and treatment temperature effects inside the thermal shrouds at the plant scale. Temperature data were collected in 20‐min intervals throughout the experiment. Temperature loggers were loosely contained in a pouch of white landscaping fabric attached to wooden stakes approximately 10 cm from the focal plant and heat packs, and 15 cm from the ground.

### Plant and caterpillar data collection

Neonate caterpillars and their host plants were first deployed to plots and measured in the morning on May 21, 2022. Plots were assessed twice a day, during morning (6:00–9:00 AM) and evening (6:00–9:00 PM) observation periods. For each observation, we visually assessed the plant status (alive, dead), percent herbivory (0%–100% leaf area damaged), caterpillar status (alive, dead, not observed), and measured caterpillar length to the nearest 0.1 mm with dial calipers. We also noted pupation, predation, and other observations. Some caterpillars were directly observed dead. Unless they were subsequently observed alive, caterpillars were also assumed to be dead if they were not observed during two consecutive intervals prior to reaching a viable pupation length. Caterpillars were determined to have pupated if pupae were observed directly, or if they were not observed during two consecutive intervals after reaching a viable pupation length (see *Analysis of pupation* *and survival*). Plant and caterpillar data collection continued twice a day until June 3, 2022, when all experimental caterpillars had either died or pupated.

### Analysis of day‐ and night‐warming

The effect of day‐ and night‐warming was modeled with a linear mixed model (package *lme4*; Bates et al., 2015). The response variable was logged temperature, day‐ and night‐warming were explanatory fixed factors, and plot id was included as a random intercepts factor to account for repeated measures. Day was defined as the hours between the morning and evening measurement periods (9:00 AM to 6:00 PM), and night was defined as the hours between the evening and morning measurement period (9:00 PM and 6:00 AM). The effects of hour or day were perfectly balanced and orthogonal across our treatments, so a model excluding those terms yielded quantitatively identical estimates of day‐ and night‐warming effects. Except where noted, model assumptions were examined and met in this and all subsequent analyses.

Using previously published thermal performance data for cabbage white caterpillars (Kingsolver, 2000), we calculated accumulated thermal exposure (i.e., degree‐days) in both the developmentally permissive thermal range (Ørsted et al., 2022) between the minimum baseline temperature (*T*_min_ = 10°C) and the developmental optimum temperature (*T*_opt_ = 35°C), and in the thermally stressful range above *T*_opt_. We modeled developmental degree‐days and thermal stress degree‐days in separate linear models with day‐ and night‐warming as explanatory factors. We also parameterized a microclimatic air temperature profile (package *TrenchR*; Buckley et al., 2023; see also Appendix S1: Section S1) using a combination of meterological and field‐collected data to confirm that our plant‐level temperatures reflected realistic variation in nature.

### Analysis of day and night herbivory

We estimated accumulated herbivore damage as the 90th quantile of all leaf area damage estimates for each plant. We used a quantile metric (instead of the maximum or last herbivory estimate) to minimize single‐observation outlier effects. We logit‐transformed this proportional measure of herbivory to better meet model assumptions (Warton & Hui, 2011), and included day‐warming, night‐warming, and their interaction as explanatory factors in a linear model. The effects of maternal line were perfectly balanced and orthogonal across treatments (*N* = 4 for each maternal line: treatment combination), so it was excluded as a factor.

We were able to separate the herbivore damage accumulated during each day and night interval by calculating the differences between subsequent morning and evening measurements. We analyzed these data using a linear mixed model with sequential observation period, treatment (C/C, C/W, W/C, W/W), day/night interval (day or night), and the interaction between treatment and day/night interval as explanatory factors. We also included a random intercepts factor for plot id to account for repeated measurements. The observation period factor accounts for the combined effects of increasing plant and caterpillar age throughout the experiment. Due to the complexity of this model, we did not attempt to separately analyze day‐ and night‐warming treatments in this model. The residuals of this model showed some minor deviations from normality (Shapiro–Wilk's *W* = 0.89), but negative and zero values prohibited log or logit transformation.

We conducted an a posteriori analysis in response to the patterns observed during exploratory plotting of day and night damage accumulation to evaluate if caterpillars feed more at night as they get older. This analysis used a more complex model including sequential observation period, treatment (C/C, C/W, W/C, W/W), day/night interval (day or night), all second‐order interaction terms, and a random intercepts factor for plot id.

### Analysis of pupation and survival

We estimated a threshold viable pupation length based on the 10th quantile of maximum attained caterpillar lengths for all caterpillars that were later observed as pupae such that 90% of known pupating caterpillars exceeded this length. We analyzed pupation as a binomial response variable in a generalized linear model with day‐warming, night‐warming, and their interaction as explanatory factors, using a logit link function.

We conducted a Cox proportional hazards model to assess the effects of day‐warming, night‐warming, and their interaction on survival (package *survival*, Therneau et al., 2024). In response to observed patterns, we also conducted an a posteriori Kaplan–Meier test to compare the survivorship of one treatment group to the others. We additionally separated day versus night mortality based on the interval when a caterpillar was last observed alive. For example, if a caterpillar was last observed alive in the morning, it was determined to have died during the day. We conducted a Pearson's χ^2^ contingency analysis to determine if the probability of mortality differed between day/night intervals, and if this difference differed by treatment.

### Analysis of caterpillar growth rates

We modeled maximum caterpillar length as the response variable in a linear model with day‐warming, night‐warming, the day‐warming:night‐warming interaction, and initial caterpillar length as explanatory factors. Maternal line was not included in this analysis because it was perfectly balanced and orthogonal across our treatments. Model residuals were bimodal, deviating somewhat from normality (Shapiro–Wilk's *W* = 0.87), but the model was analyzed without modification. In this case, this pattern likely reflects early mortality in a subset of caterpillars, and a statistical correction to improve residual normality (e.g., by including mortality as a factor in the model) would obscure a biological mechanism of interest.

We quantified caterpillar growth rates as the mean change in caterpillar length per day for the subset of caterpillars that survived to day 8 (i.e., the absolute growth rate, AGR_8_ = (length_8_ – length_0_)/8). AGR (as opposed to a relative growth rate) is appropriate in situations where growth is approximately linear. The 8‐day window used for this growth rate calculation represents a balance between increased sample size with shorter windows versus increased time averaging with longer windows, and exploratory plotting with a shorter 4‐day window showed qualitatively similar results.

We analyzed a linear model with AGR_8_ as the response variable, and day‐warming, night‐warming, the day‐warming:night‐warming interaction, maternal line and initial caterpillar length as explanatory factors. In this analysis, maternal line was not perfectly balanced and orthogonal in this analysis because only a subset of caterpillars survived to day 8. Maternal line was initially included in this model as a fixed covariate to account for potential differences between maternal lines, and because it has too few levels (*N* = 5) to be meaningfully included as a random factor. Initial caterpillar length was also initially included as a covariate to account for potential differences due to pre‐manipulation size. However, both maternal line and initial length did not contribute significantly to preliminary models and were excluded from the final analysis.

To evaluate an a posteriori hypothesis that emerged from exploratory plotting, we modeled caterpillar growth increments during day and night intervals with a linear mixed model using individual, day‐ or night‐specific growth increments (measured changes in caterpillar length) as the response variable. This model included sequential observation period, treatment (C/C, C/W, W/C, W/W), day/night interval (day or night), and the interaction between treatment and day/night interval as fixed factors, and also included a random intercepts factor for plot id to account for repeated measurements.

## RESULTS

### Day‐ and night‐warming

**FIGURE 1:**
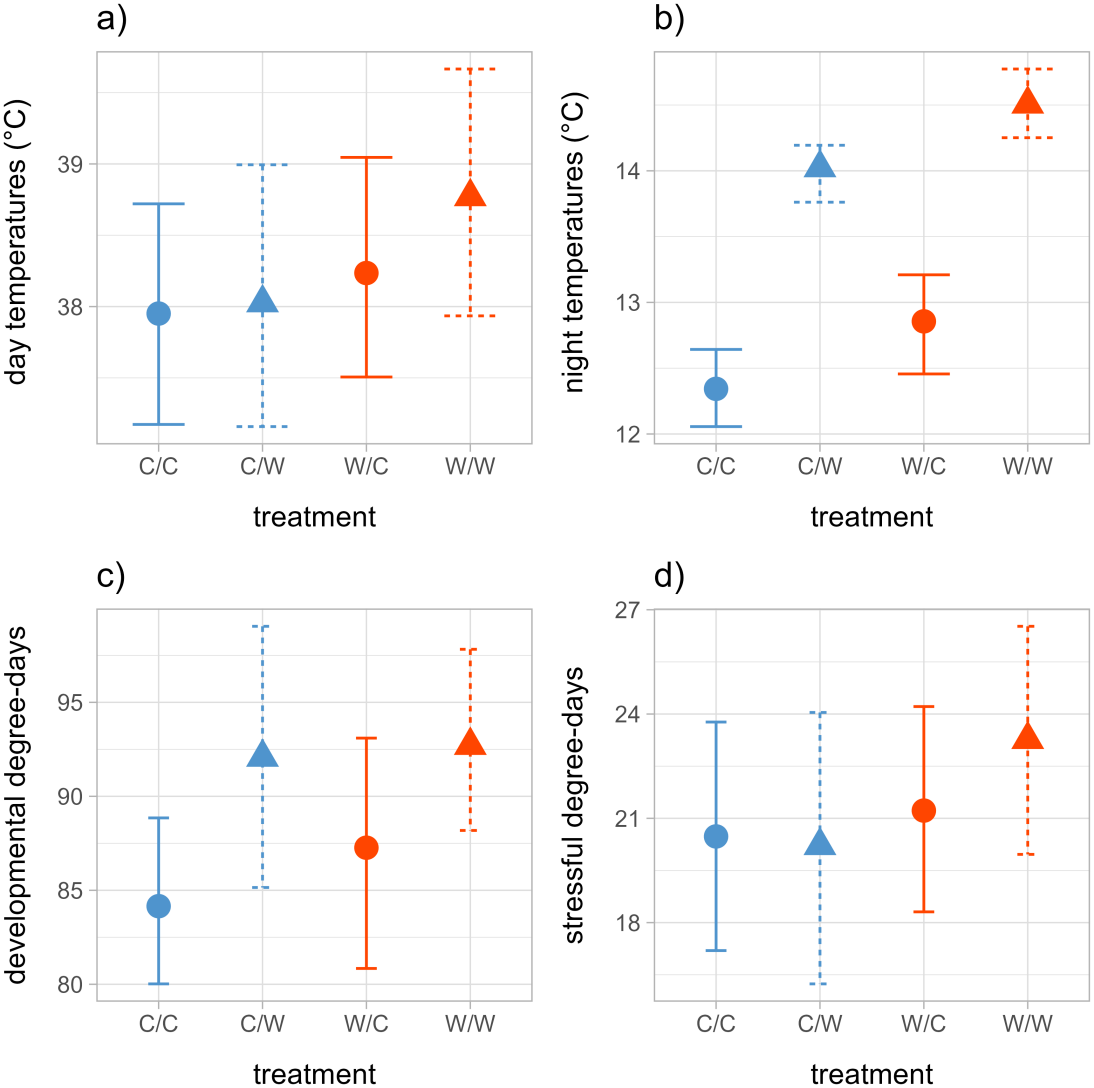
Mean (a) daytime and (b) nighttime temperatures measured throughout this experiment. (c) Accumulated degree‐days in the developmentally permissive range (10–35°C) and (d) in the thermally stressful range (>35°C). Color indicates day‐warming treatment (red is day‐warmed, blue is control) and line type and point shape indicate night‐warming treatment (dotted line and triangle is night‐warmed, solid line and circle is control). Error bars represent 95% CI. C/C, control; C/W, night‐warming only; W/C, day‐warming only; W/W, day‐ and night‐warming.

On average, day‐warmed replicates were 0.5°C warmer during the day (38.5 vs. 38.0°C, χ^2^(1) = 1.4, *p* = 0.242, Figure 1a), and night‐warmed replicates were 1.7°C warmer during the night (14.3 vs. 12.6°C, χ^2^(1) = 51.4, *p* < 0.001, Figure 1b) compared with replicates that did not receive day‐ or night‐warming respectively. There was some evidence that day‐warming also affected nighttime temperatures and vice versa (Table 1; Appendix S1: Figures S2 and S3), consistent with thermal carryover between day and night. Day‐warming increased nighttime temperatures by 0.5°C (χ^2^(1) = 9.3, *p* = 0.002) and night‐warming increased daytime temperatures by 0.32°C (χ^2^(1) = 0.5, *p* = 0.464). Across a mean 24‐h period, the effects of day‐warming were most apparent in midafternoon (between 1:00 PM and 3:00 PM), whereas the effects of both day‐ and night‐warming were greatest at the beginning of the night and persisted until the early morning (around 6:00 AM; Appendix S1: Figure S2).

**TABLE 1:** Summary of mean day and night temperatures, development rates, survival rates, and behavioral differences between treatment groups.

Treatment	Mean temperature (°C)		Development			Survival		Behavior	
	Day	Night	Growth (mm/day)	Mean length (mm)	Nocturnal growth (%)	Mortality (%)	Diurnal mortality (%)	Herbivory (%)	Nocturnal herbivory (%)
C/C	38.0	12.3	1.5	12.8	27	60	83	14	90
C/W	38.0	14.0	2.0	9.0	21	80	87	7	66
W/C	38.2	12.9	1.5	11.3	35	75	80	10	53
W/W	38.8	14.5	1.7	13.0	46	65	92	12	126

Abbreviations: C/C, control; C/W, night‐warming only; W/C, day‐warming only; W/W, day‐ and night‐warming.

Night‐warming increased developmental degree‐day exposure by 6.6 degree‐days across this 14‐day experiment (Figure 1c; *F*_1,31_ = 4.6, *p* = 0.04). Night‐warming did not significantly increase thermal stress exposure (Figure 1d; 0.96 degree‐days, *F*_1,31_ = 0.28, *p* = 0.60), and day‐warming did not significantly increase thermal exposure in either the developmental (Figure 1c; 1.87 degree‐days, *F*_1,31_ = 0.37, *p* = 0.55) or stressful range (Figure 1d; 1.90 degree‐days, *F*_1,31_ = 1.1, *p* = 0.31).

At the plant scale, measured temperatures varied widely between daily minimum and maximum values (Appendix S1: Figures S3 and S4a). By comparison, weather station air temperatures showed substantially lower maximum temperatures and less daily variation (Appendix S1: Figure S4a). Observed differences between plant‐scale temperatures (at approximately 20 cm height) and weather station temperatures (at 1.25–2 m height, WMO, 2023) were consistent with air temperature profiles estimated from existing microclimatic models (Appendix S1: Section S1, Figure S5; Buckley et al., 2023; Kearney & Porter, 2017).

### Day and night herbivory

**FIGURE 2:**
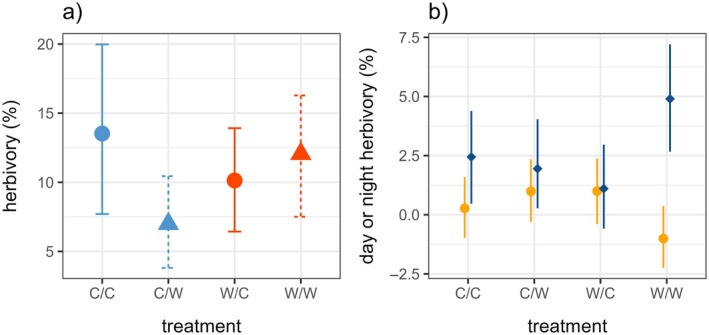
(a) The accumulated percentage of herbivory by treatment (90% percentile of all observations). Color indicates day‐warming treatment (red is day‐warmed, blue is control) and line type and point shape indicate night‐warming treatment (dotted line and triangle is night‐warmed, solid line and circle is control). (b) Mean day and night increments of percentage of herbivory by treatment. Daytime points are orange circles and nighttime points are dark blue diamonds. Error bars represent 95% CI. C/C, control; C/W, night‐warming only; W/C, day‐warming only; W/W, day‐ and night‐warming.

Accumulated herbivory was lowest in the night‐warming only (C/W) treatment (7.0%, Figure 2), compared to damage percentages of 10%–14% in the other treatments (Table 1). The day‐warming:night‐warming interaction was not significant (*F*_1,76_ = 1.3, *p* = 0.25). However, because the direction of the night‐warming effect differed with and without day‐warming, the main effects largely balanced each other out (Figure 2a; day‐warming: *F*_1,77_ = 0.58, *p* = 0.45; night‐warming: *F*_1,77_ = 0.50, *p* = 0.48).

Most herbivore damage accumulated during the night than during the day (1.99% per 12 h interval, χ^2^(1) = 13.2, *p* < 0.001, Figure 2b, Table 1). In the C/C group, 90% of the total damage accumulation occurred at night, compared with 66% in the C/W group and 53% in the W/C group. In the W/W group, damage increased by an average of 4.9% each night and actually decreased by 1% each day, indicative of compensatory plant growth during the day. Effectively, this means that 100% of the herbivory in the W/W group occurred at night (Figure 2b). These differences were highly significant (treatment:day/night interaction, χ^2^(3) = 13.5, *p* = 0.004). Leaf damage increased with sequential observation period (Appendix S1: Figures S4b and S6; 0.19% per 12 h interval, χ^2^(1) = 14.1, *p* < 0.001), consistent with increasing caterpillar size. We also observed a significant interaction between the observation period and the day/night interval (χ^2^(1) = 124.5, *p* < 0.001), consistent with our a posteriori hypothesis that caterpillars feed more at night as they get older (Appendix S1: Figures S4b).

### Pupation and survival

The threshold for viable pupation length was estimated to be 18 mm, based on the 10th quantile of attained lengths for all caterpillars that were known to have pupated, and a natural break in the histogram of attained caterpillar lengths (Appendix S1: Figure S7).

Night‐warming appeared to decrease pupation in the absence of day‐warming but showed the opposite pattern in the presence of day‐warming (Appendix S1: Figure S8). This interaction was marginal in a binomial generalized linear model of pupation (χ^2^(1) = 2.2, *p* = 0.14).

All caterpillars attained a viable pupation size or were determined to have died during this experiment (e.g., we did not have any censored survivorship data). Evidence of direct predation was directly observed in *N* = 3 cases (one crab spider [Thomisidae], one unknown spider, and one unknown predator), and pupation was directly observed in *N* = 7 cases (including one that was observed being eaten by ants after pupation). In *N* = 17 other cases, the caterpillar exceeded the viable pupation threshold and was assumed to have pupated. In the remaining *N* = 53, the caterpillar never exceeded the viable pupation threshold and was assumed to have died (Appendix S1: Figure S9).

**FIGURE 3:**
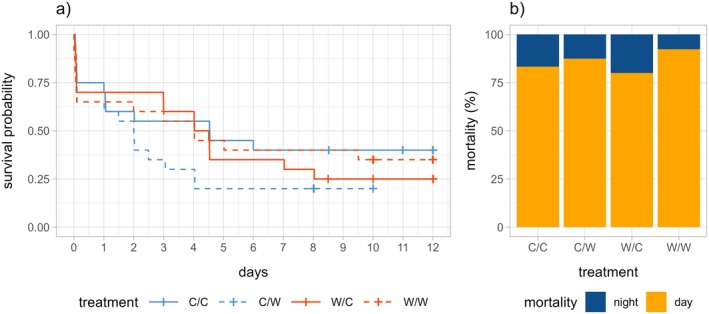
(a) A Kaplan–Meier survivorship plot by treatment. Color indicates day‐warming treatment (red is day‐warmed, blue is control) and line type indicates night‐warming treatment (dotted line is night‐warmed, solid line is control). Crosses represent pupation. (b) A stacked barplot of day and night mortality by treatment. C/C, control; C/W, night‐warming only; W/C, day‐warming only; W/W, day‐ and night‐warming.

We did not observe significant overall interaction (day‐warming:night‐warming, *p* = 0.21) or main effects on survival (day‐warming, *p* = 0.77, night‐warming, *p* = 0.39). However, survivorship curves suggested lower early survival in the night‐warming only (C/W) group (Figure 3a, Table 1), and an a posteriori comparison between the C/W group and all other treatments suggested that this contrast was marginally significant (χ^2^(1) = 2.0, *p* = 0.15). In addition, we did observe that most mortality (86%) occurred during the day (Figure 3b, Pearson's χ^2^(1) = 28.6, *p* < 0.001). This day‐biased mortality was relatively consistent across treatment groups (Pearson's χ^2^(3) = 0.96, *p* = 0.81), with the proportion of mortality that occurred during the day varying between 80% in the W/C group and 92% in the W/W group (Table 1).

### Caterpillar growth rates

**FIGURE 4:**
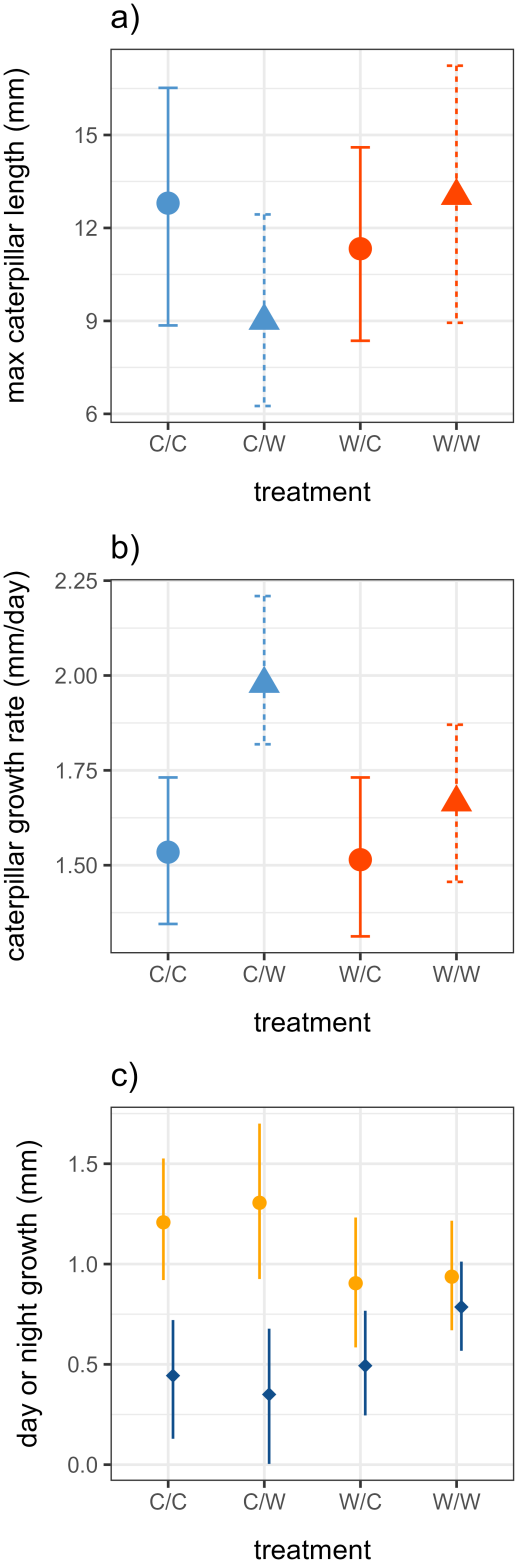
(a) Maximum measured caterpillar length by treatment. Color indicates day‐warming treatment (red is day‐warmed, blue is control) and line type and point shape indicate night‐warming treatment (dotted line and triangle is night‐warmed, solid line and circle is control). (b) Absolute growth rate at 8 days (AGR_8_) by treatment. Colors and symbols as in panel (a). (c) Mean day and night increments of caterpillar growth by treatment. Daytime points are orange circles and nighttime points are dark blue diamonds. Error bars represent 95% CI. C/C, control; C/W, night‐warming only; W/C, day‐warming only; W/W, day‐ and night‐warming.

The max lengths of caterpillars in the night‐warming only (C/W) treatment were 9.0 mm, compared with 12.8 mm in the C/C group, 11.3 mm in the W/C group, and 13.0 mm in the W/W group (Figure 4a). The interaction of day‐warming and night‐warming was marginal (*F*_1,75_ = 2.00, *p* = 0.16), and we did not detect any significant main effects of day‐warming or night‐warming on maximum caterpillar length (Figure 4a).

The interaction between day‐warming and night‐warming did not significantly affect growth rates (Figure 4b, *F*_1,22_ = 1.5, *p* = 0.24) and was removed from the analysis. In a simplified model, night‐warming increased caterpillar growth rates by 0.28 mm/day (Figure 4b, *F*_1,23_ = 5.2, *p* = 0.03) and day‐warming reduced caterpillar growth by 0.15 mm/day (Figure 4b, *F*_1,23_ = 1.5, *p* = 0.24). On average, caterpillar growth rates were 29% higher in the C/W treatment compared with the control group (Figure 4b and Appendix S1: Figure S11), and this difference was significant in an a posteriori contrast (*F*_1,10_ = 6.8, *p* = 0.03).

On average, 34% of caterpillar growth occurred during the night (Figure 4c; Appendix S1: Figure S4c). In the C/C and C/W groups, nocturnal caterpillar growth accounted for 27% and 21% of the 24‐h mean growth increment respectively. In the day‐warmed W/C and W/W groups, the proportion of caterpillar growth occurring at night increased to 35% and 46% respectively. Growth increments increased slightly with observation interval (Appendix S1: Figure S4c; χ^2^(1) = 39.4, *p* < 0.001), but the small effect size was consistent with very slowly accelerating, nearly linear growth over development (Appendix S1: Figure S10). The interaction between treatment and day/night interval was also significant (χ^2^(3) = 8.6, *p* = 0.035), consistent with the differences in day‐ and night‐specific growth increments observed across the treatment groups (Figure 4c).

## DISCUSSION

We first discuss our experimental warming manipulation, then synthesize our key findings before reviewing our results in the context of past work and broader questions.

### Experimental day‐ and night‐warming at the plant scale

Heat packs effectively warmed the area immediately around individual caterpillars and their host plants and allowed us to experimentally separate the effects of day‐ versus night‐warming in a 2 × 2 factorial design. Although each heat pack generated the same amount of heat, the observed effects on air temperature were much larger at night than during the day (Figure 1). This difference could result from radiant solar warming during the day potentially minimizing the relative contributions of the heat packs, or from increased convective heat loss due to slightly higher windspeeds during the day (0.43 vs. 0.29 m/s; Appendix S1: Section S1).

The observation that day‐warming also increased nighttime temperatures (Figure 1b; Appendix S1: Figure S2) suggests the possibility of a diel carryover effect, possibly by warming or drying the plant/soil system. The magnitude and duration of this carryover effect was surprisingly large, with measurable day‐warming effects persisting throughout most of the night (Appendix S1: Figure S2). This finding suggests that increased daytime temperatures could also further increase night‐warming at the plant scale.

### The complex effects of warming on herbivory, growth and survivorship

The results of this study may initially appear puzzling: how could the night‐warming only (C/W) conditions that resulted in the highest rates of caterpillar growth (Figure 4b) also cause the highest early mortality (Figure 3a), the shortest caterpillar lengths (Figure 4a), the least accumulated herbivory (Figure 2a) and the lowest rates of pupation (Appendix S1: Figure S8)?

The night‐warming only treatment (C/W) showed high developmental degree‐day accumulation (Figure 1c), suggesting that this treatment created temperatures that were conducive to rapid growth. The caterpillars in this group also showed somewhat day‐shifted herbivory (Figure 2b) and growth (Figure 4c). This treatment did not increase the diel thermal range (Table 1), so these results are not explained by the general expectation of increasing energetic costs associated with greater fluctuations across the diel cycle (Colinet et al., 2015). However, because ectotherms generally show a limited ability to prevent the increased metabolic demands that are associated with higher temperatures (Kern et al., 2015; Weaving et al., 2022), we suggest that the costs of rapid growth in this context may have depleted internal resources, or required increased daytime foraging, potentially exposing caterpillars to greater risks from diurnal enemies and thermal stress. The increased nighttime temperatures may also have limited opportunities for thermal repair against this backdrop of rapid growth. These results suggest that the same thermal conditions that maximized growth rates may have also had the largest negative effects on survivorship.

By comparison, the treatment combining day‐ and night‐warming (W/W) did not show strongly reduced survival (Figure 3a) or particularly fast growth (Figure 4b), despite also experiencing high developmental degree‐day accumulation (Figure 1c). This treatment experienced higher thermal stress accumulation in theory (Figure 1d) but showed strongly reduced daytime herbivory (Figure 2b) and growth (Figure 4c). Overall, thermal stress in the W/W treatment did not strongly limit growth and survivorship as we had expected. On the contrary, the strong shift toward increased nighttime activity in this group (Figures 2b and 4c) may have slowed growth but increased survivorship compared with the C/W treatment. Both biotic (e.g., predation risk) and abiotic (e.g., thermal) factors could have contributed to this pattern, and this treatment suggests the importance of behavioral changes in diel activity on development and survival.

### Conclusions

Previous lab studies (e.g., Bai et al., 2019; Ma et al., 2021; Zhao et al., 2014) and broader patterns like the temperature‐size rule (Angilletta et al., 2004) or the slow growth‐high mortality hypothesis (Benrey & Denno, 1997) cannot fully explain the results that we observed in this field study. Our findings are more complex than our initial expectations, but provide support for the general idea that periods of reduced activity may be important for successful development (Bai et al., 2019; Braby & Lyonns, 2003; Colinet et al., 2015; Kingsolver, 2000; Kingsolver et al., 2015, 2021; Ma et al., 2017; Nail et al., 2015). Our results are also consistent with the hypothesis that warmer nighttime conditions could limit a temporal thermal refuge for caterpillars (Gaston, 2019; Speights et al., 2017), with accumulating negative effects that do not require increased exposure to acutely stressful temperatures (Colinet et al., 2015; Ma et al., 2017; Speights et al., 2017; Zhao et al., 2014). Thermal restrictions on foraging time further suggest the possibility of a “metabolic meltdown” (Huey & Kingsolver, 2019) where increasing temperatures simultaneously increase metabolic rates and decrease food availability.

This study illustrates both the value and some of the challenges associated with day‐ and night‐specific warming experiments in the field. Importantly, we suggest that the results that we observed in this experiment are unlikely to have emerged without the complexity of a field experiment. In addition to the direct physiological effects of day‐ and night‐warming, we observed changes in diel activity and survivorship that may reflect the consumptive and nonconsumptive effects of predators in this system, consistent with a previous field study that manipulated day‐ and night‐warming (Barton & Schmitz, 2018). Whereas Barton and Schmitz (2018) focused on effects at the community level (e.g., herbivore and predator behavior, and plant diversity and biomass in mesocosms), our study focused on the behavior, growth, and survival of a focal herbivore species in an open community. Future experimental field studies are likely to improve our understanding of the complex ways in which real‐world systems are likely to respond to anthropogenic changes in day‐ and night‐warming. Whereas lab experiments can provide valuable insights, field experiments that manipulate day and night temperatures across development, conducted in natural microhabitats exposed to realistic diel variation with free‐living organisms and open access to a broader community can also provide unique insights into how ectotherms will respond to a warming climate.

## CONFLICT OF INTEREST STATEMENT

The authors declare no conflicts of interest.

## Supporting information


Appendix S1.

## Data Availability

Data and code (Yang et al., 2025) are available in Dryad at https://doi.org/10.5061/dryad.n8pk0p35s.
