# Supplementary material for: Live fast, die young? Day‐ and night‐warming affect the growth, survivorship, and behavior of caterpillars in the field
Source: Ecology. 2025 Jul 3;106(7):e70150. doi: 10.1002/ecy.70150 (PMC12223705; doi:10.1002/ecy.70150)
Supplement: Supplementary file 1 — Appendix S1. [file ECY-106-e70150-s001.pdf]

Appendix S1

Live fast, die young? Day- and night-warming affect the growth, survivorship, and behavior of caterpillars in the field

Louie H. Yang, Elizabeth G. Postema, Heran Arefaine, Fernanda Y. Cohoon, Emma A. Deen, Yvonne L. Durand, Gwendolyn I. Erdosh, Hailey Ma, Courtney N. Mausling, Sarah Solís, Madeline R. Wilson

Ecology

Figure S1. Photographs of the a) field site grid and b) a close-up of a thermal shroud. Photographs by L.H. Yang.

a)

b)

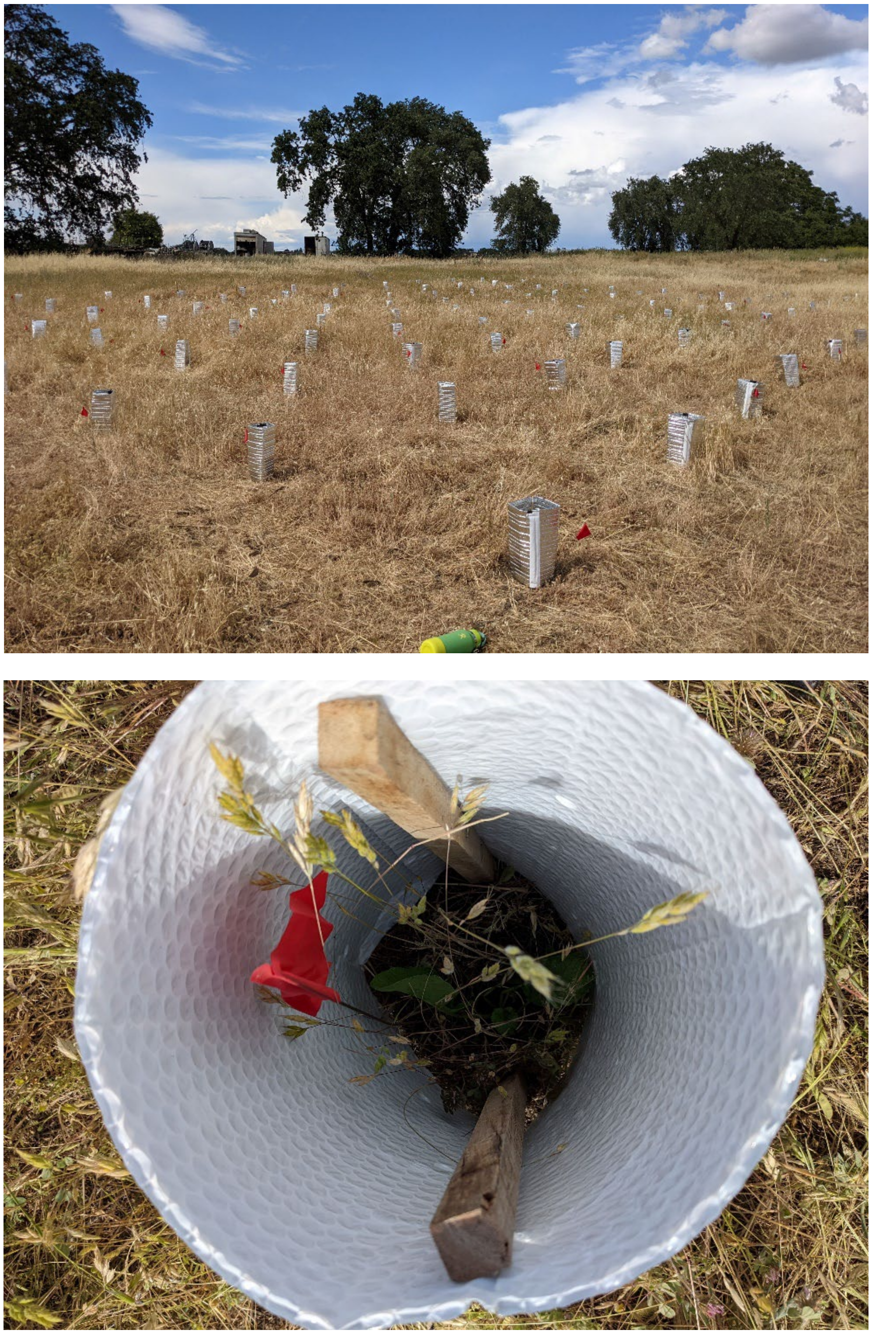

Figure S2. Mean hourly temperatures across the experiment. Points represent the hourly mean of 8 or 9 iButtons in each treatment, error bars represent 95% CI. Color indicates day-warming treatment (red is day-warmed, blue is control) and line type and point shape indicates night-warming treatment (dotted line and triangle is night-warmed, solid line and circle is control). There is a relatively small effect of daytime warming and a relatively large effect on nighttime warming. Daytime temperatures showed much greater variability than nighttime temperatures.

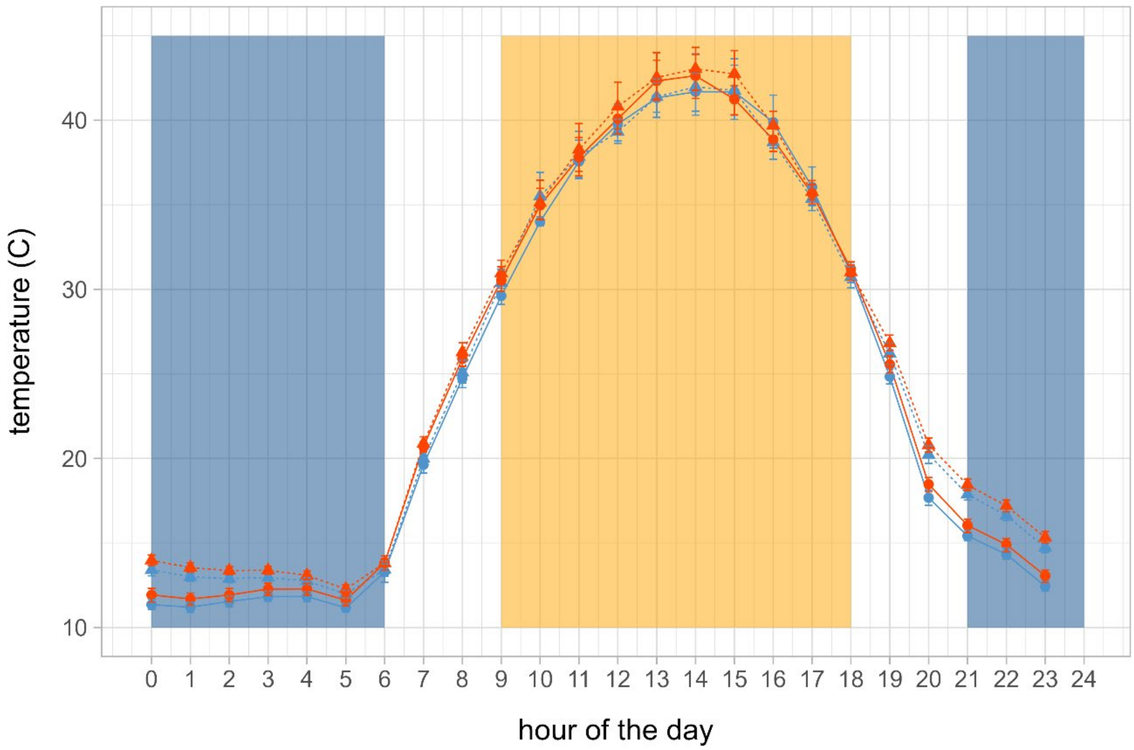

Figure S3. Temperature variation across this experiment by treatment. Each line represents an individual iButton. Daytime intervals between 9AM and 6PM are shown with a red line and orange background. Nighttime intervals between 9PM and 6AM are shown with a blue line and dark blue background.

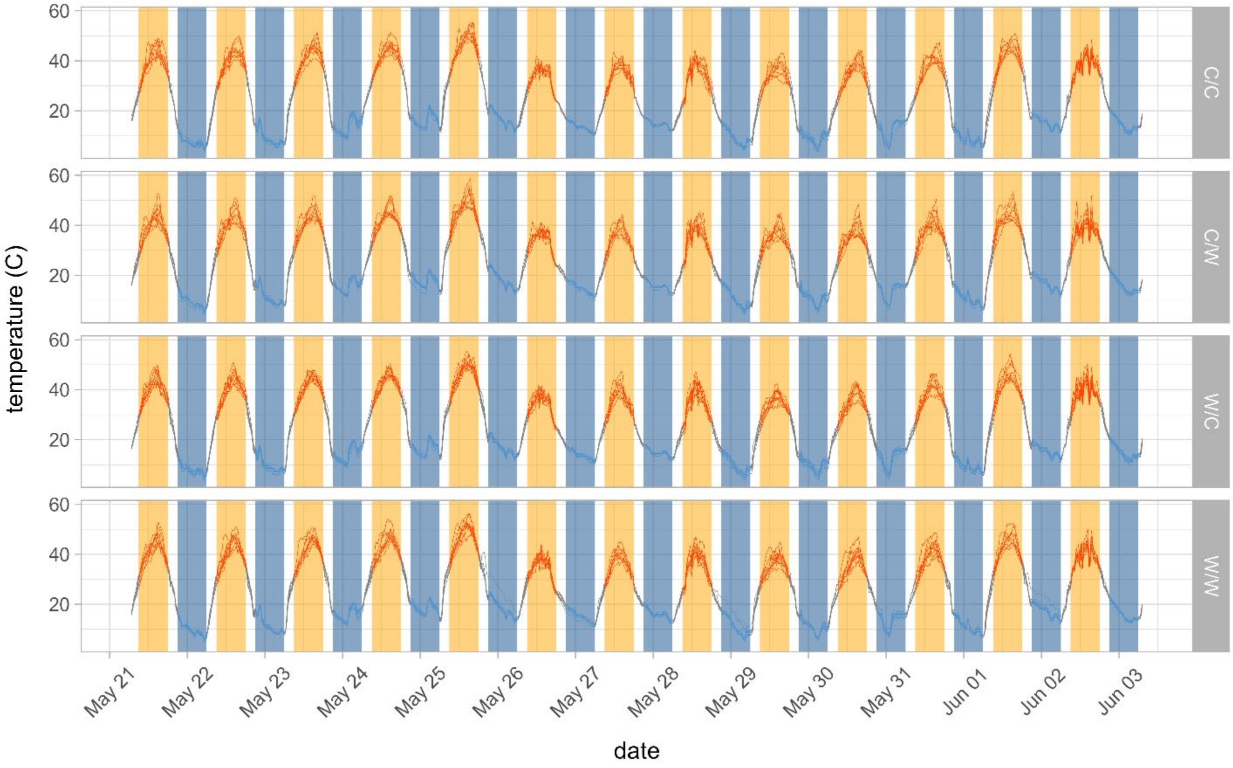

Section S1: Supplemental Methods

We were initially concerned that the observed differences between weather station air temperature measurements and our plant-scale measurements (Figure 3a) could have been an artifact of the thermal shrouds used in this experiment. However, parameterized microclimatic models predicted steep thermal profiles near the ground that were consistent with our observed differences between plant-level temperatures and nearby weather station temperatures, suggesting that the thermal shrouds in this experiment likely did not result in unrealistic plant-level temperatures.

To assess this observed difference between plant-scale temperatures and weather station air temperatures in the context of existing microclimatic models, we parameterized a model to estimate two air temperature profiles between the ground surface and 2 m using the *air_temp_profile()* function in the *TrenchR* package (Buckley et al. 2023), which itself uses a routine from the *NicheMapR* package (Kearney and Porter 2017). We fit this function with the following parameters:

parameter	description	profile 1	profile 2
T_r	air temperature (C) at reference height.	41.2	39.4
u_r	windspeed (m s^{-1}) at reference height.	0.4	0.4
z_r	initial reference height (m).	1	1
z_0	surface roughness (m).	0.04	0.04
z	height to scale (m).	0 to 2	0 to 2
T_s	surface temperatures (C).	53.3	66.5

The parameters T_r and T_s represented the daily maximum 1 m air temperature and ground surface temperatures respectively, measured without any thermal shroud at the same field site, on August 27, 2024 using two multi-channel thermocouple loggers (HOBO, Onset Computer) approximately 5 m apart.

The windspeed parameter was estimated from the 30-year average daytime windspeed during the period of this experiment (May 21 to June 3) using the U.S. Climate Hourly Normals dataset (Arguez et al. 2010) from the nearest available weather station (USW00023232, SACRAMENTO EXECUTIVE AIRPORT, CA US).

Surface roughness is defined as the height above the ground at which the wind speed would be expected to fall to zero, and was estimated using common values for a medium height grassland with some nearby trees (Wieringa 1986).

In Appendix S1: Figure S5, we plot the mean and SD of daily maximum temperatures from the DAVIS 2 WSW EXPERIMENTAL FARM, CA US weather station (USC00042294) 750 m from our field site (NOAA 2024) assuming a sensor height of 1.625 m, at the midpoint of the standard weather station height range of 1.25-2 m (WMO 2023).

Figure S4. a) Daily min and max temperature variation across the experiment for plant-scale temperature loggers (filled points) and a nearby weather station (open points). b) Mean daily day and night increments of % herbivory across the experiment. c) Mean daily day and night increments of caterpillar growth across the experiment. Error bars represent 95% CI. Daytime points are shown in orange circles and nighttime points are shown in dark blue diamonds.

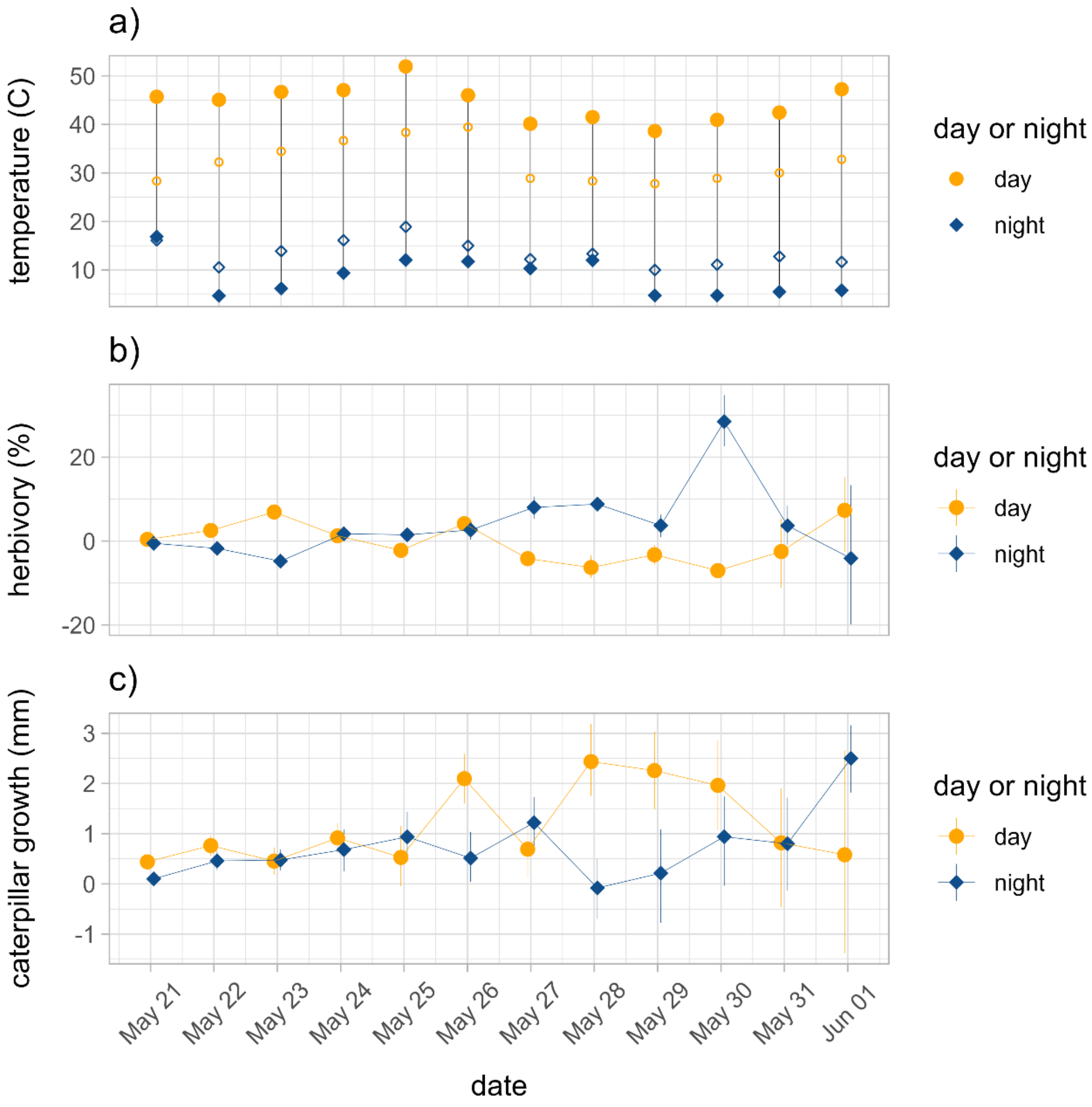

Figure S5. The black points show simultaneous temperature measurements at 0 and 1 m at two locations within our field site measured without thermal shrouds on August 27, 2024. These points were used to parameterize a microclimate air temperature profile (Appendix S1: Section S1). We compared these curves with the daily maximum plant-scale temperature measurements from our experiment (red and orange filled points) and from a nearby weather station (red and orange open points) on two experimental days with comparable temperature conditions (May 25, 2022 in red and May 26, 2022 in orange). For the experimental plant-scale temperatures, points and error bars represent the mean and SD of daily maximum temperatures recorded by iButtons in the unwarmed C/C plots. For the experimental weather station temperatures, points and errorbars represent the maximum daily temperatures and the standard height range (1.25-2 m) of weather station measurements (WMO 2023). These results suggest that the observed differences between weather station measurements and the plant-scale measurements during our experiment are consistent with the expected microscale thermal profile in the absence of thermal shrouds.

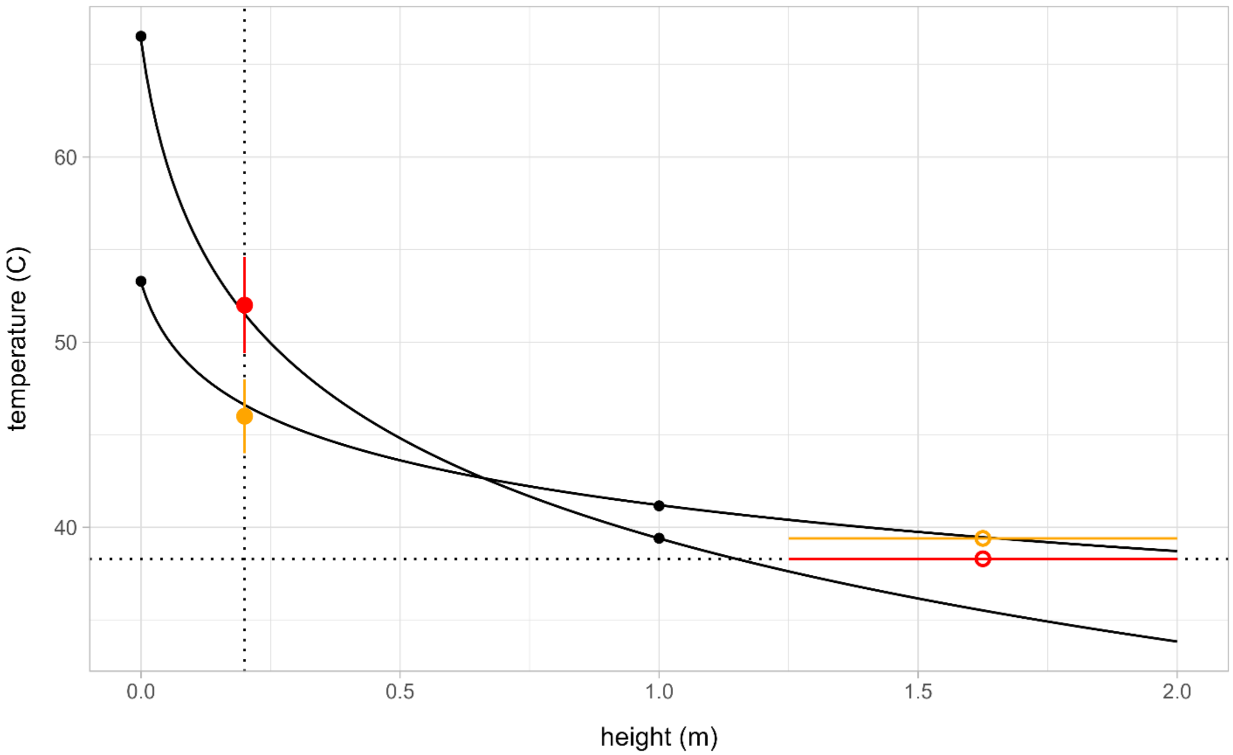

Figure S6. Observed percent herbivore damage for each plant with a live caterpillar present. Day-warming is shown in red. Color indicates day-warming treatment (red is day-warmed, blue is control) and line type and point shape indicates night-warming treatment (dotted line and triangle is night-warmed, solid line and circle is control). Points indicate the last observation of each caterpillar alive.

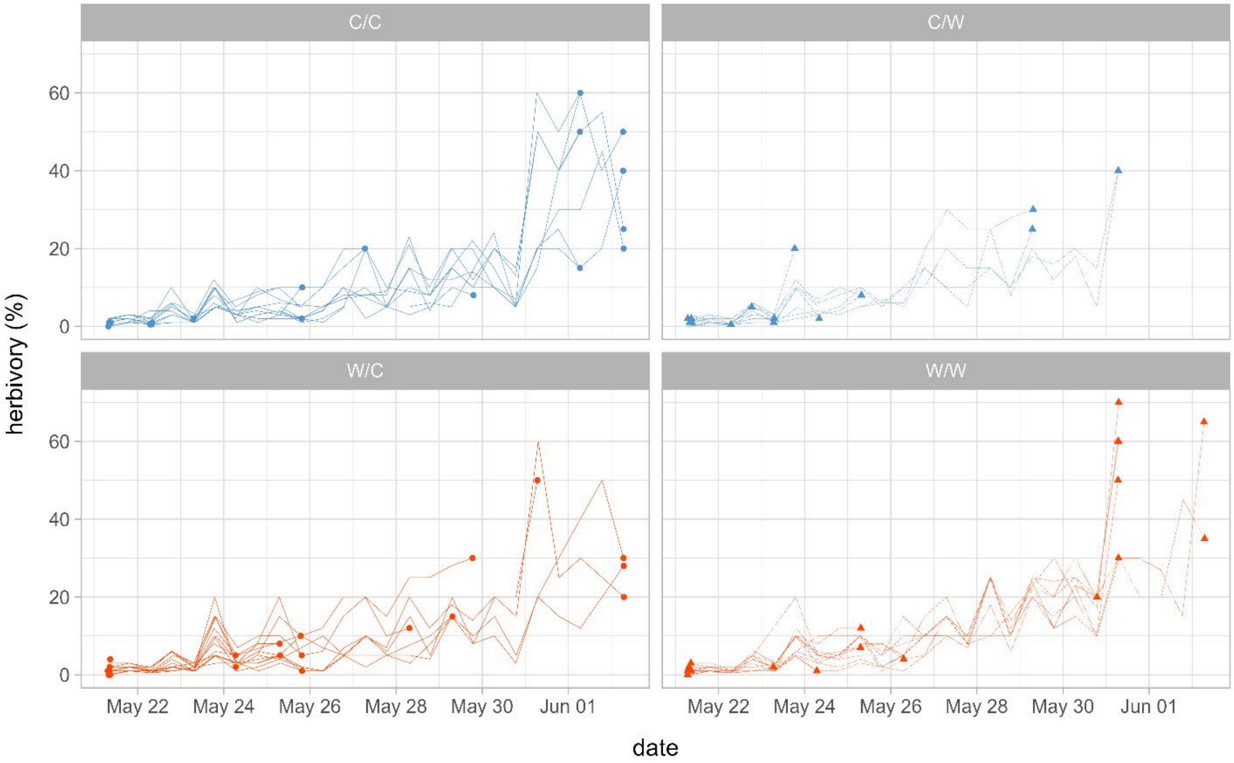

Figure S7. Histogram of maximum observed caterpillar lengths. Most caterpillars probably left the host plant prior to pupation, so pupation was directly observed only in a subset of cases. The 10th quantile of the maximum lengths for caterpillars where pupation was directly observed was 18mm.

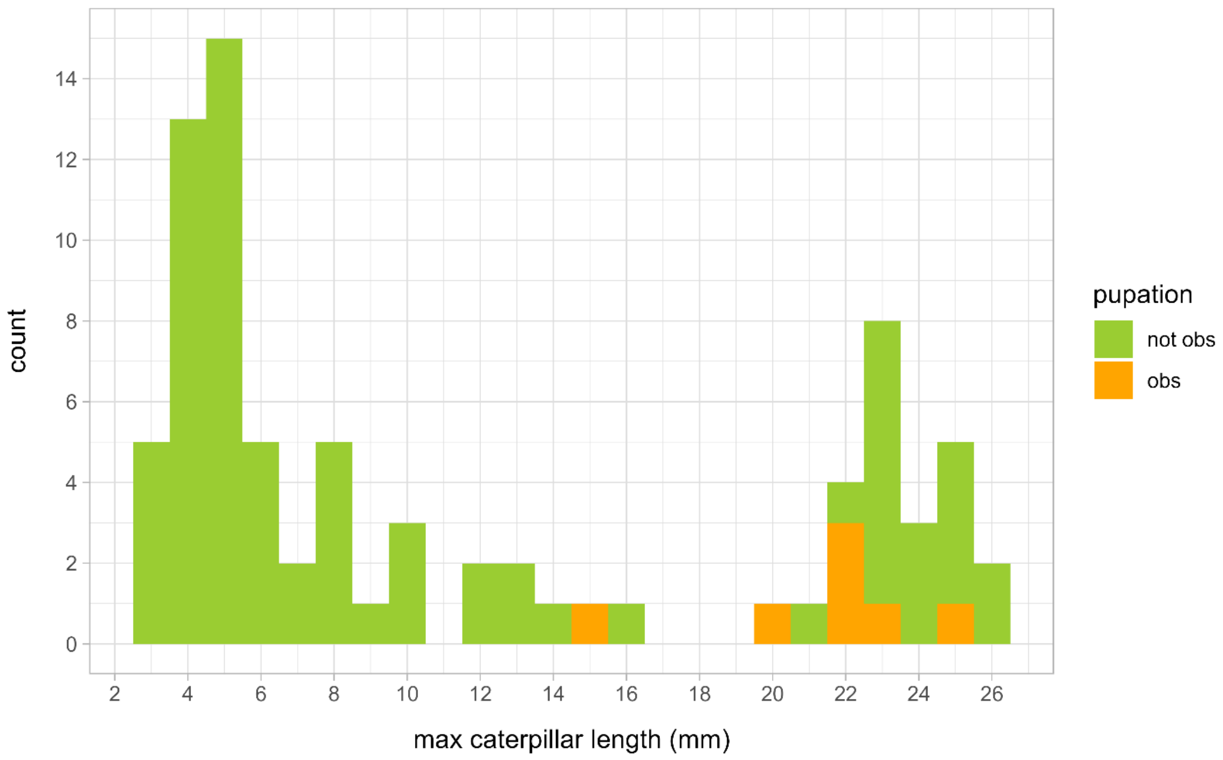

Figure S8. Estimated pupation rates by treatment. Color indicates day-warming treatment (red is day-warmed, blue is control) and point indicates night-warming treatment (triange is night-warmed, circle is control).

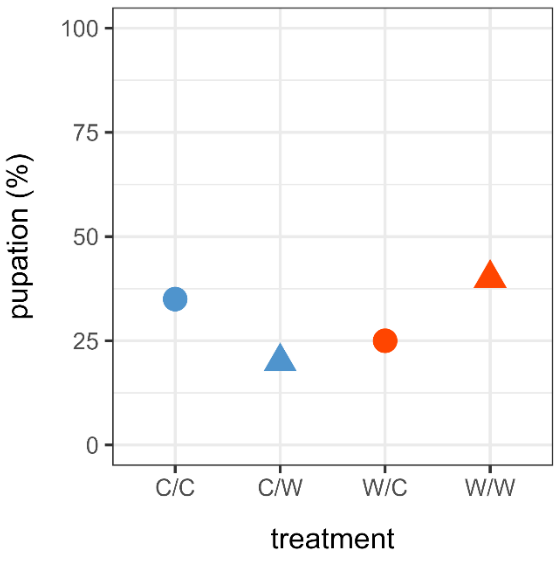

Figure S9. The observation status of individual caterpillars throughout this experiment.

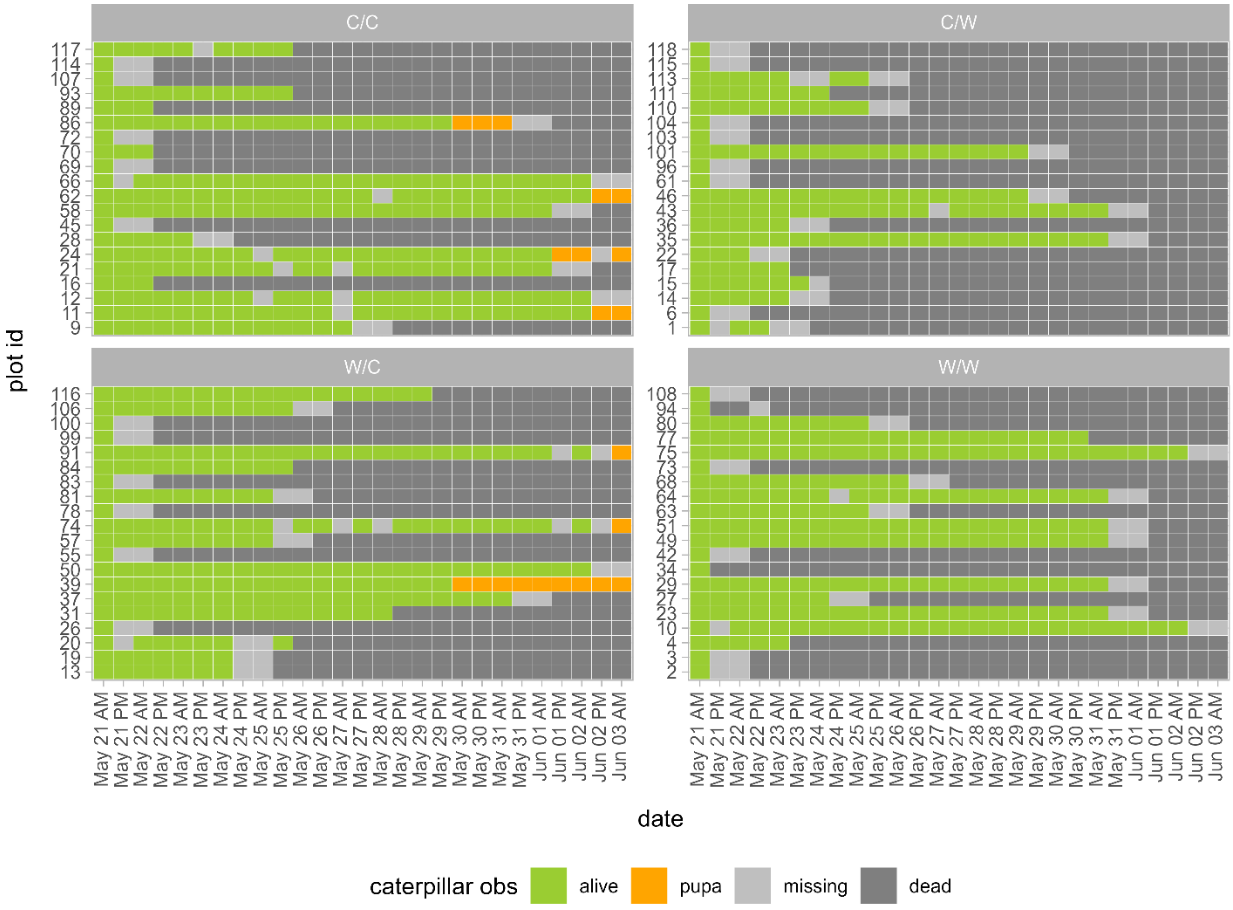

Figure S10. Observed individual caterpillar lengths (mm). Day-warming is shown in red. Color indicates day-warming treatment (red is day-warmed, blue is control) and line type and point shape indicates night-warming treatment (dotted line and triangle is night-warmed, solid line and circle is control). Points indicate the last observation of each caterpillar alive.

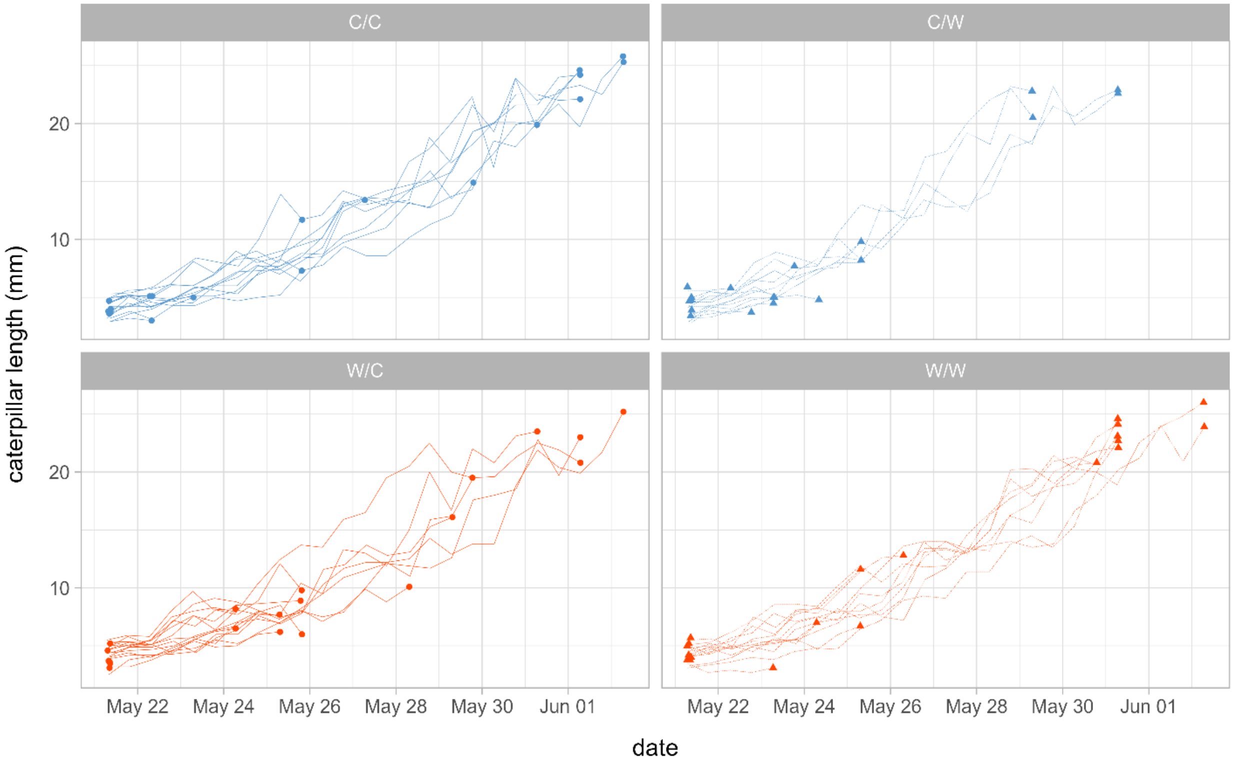

Figure S11. Mean caterpillar length by treatment. Day warming is shown in red, and night-warming is shown with dashed lines.

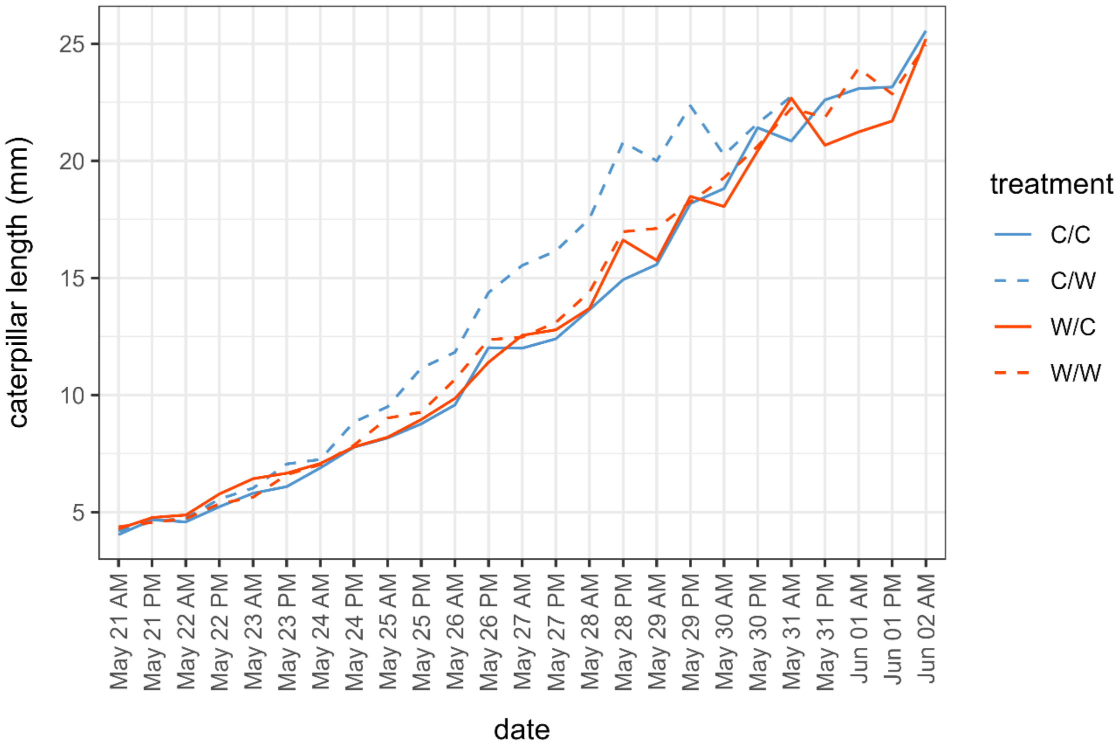

References

Arguez, A., I. Durre, S. Applequist, M. Squires, R. Vose, X. Yin, and R. Bilotta. 2010. U.S. Climate Normals Product Suite (1981-2010). NOAA National Climatic Data Center.

Buckley, L., B. B. Ortiz, I. Caruso, A. John, O. Levy, A. Meyer, E. Riddell, Y. Sakairi, J. Simonis, and B. Helmuth. 2023, September 13. TrenchR: Tools for Microclimate and Biophysical Ecology.

Kearney, M. R., and W. P. Porter. 2017. NicheMapR – an R package for biophysical modelling: the microclimate model. Ecography 40:664–674.

NOAA. 2024. Climate Data Online. https://www.ncdc.noaa.gov/cdo-web/.

Wieringa, J. 1986. Roughness-dependent geographical interpolation of surface wind speed averages. Quarterly Journal of the Royal Meteorological Society 112:867–889.

WMO. 2023. Guide to Instruments and Methods of Observation. World Meteorological Organization, Geneva.
